# Development of a Metaphyseal Non-Union Model in the Osteoporotic Rat Femur

**DOI:** 10.3390/bioengineering10030338

**Published:** 2023-03-07

**Authors:** Amelie Deluca, Andrea Wagner, Bettina Faustini, Nadja Weissenbacher, Christian Deininger, Florian Wichlas, Herbert Tempfer, Ernst J. Mueller, Andreas Traweger

**Affiliations:** 1 Institute of Tendon and Bone Regeneration, Spinal Cord Injury & Tissue Regeneration Center Salzburg, 5020 Salzburg, Austria; 2 Department of Traumatology, KABEG—Klinikum Klagenfurt am Woerthersee, 9020 Klagenfurt, Austria; 3 Austrian Cluster for Tissue Regeneration, 1200 Vienna, Austria; 4 Department of Orthopedics and Traumatology, Salzburg University Hospital, Paracelsus Medical University, 5020 Salzburg, Austria

**Keywords:** rat models for fracture healing, osteoporosis, metaphyseal fracture, pseudoarthrosis

## Abstract

The aim of this current study was to establish a metaphyseal femoral non-union model in osteoporotic rats by comparing a power tool versus a manual tool for fracture creation. Twelve adult female Sprague Dawley rats were ovariectomized (OVX) and received a special diet for 6 weeks. Biweekly pQCT measurements confirmed a significant reduction in the cancellous and total bone mineral density in OVX rats compared to control (CTRL) animals. After 6 weeks, OVX rats underwent surgery creating a distal metaphyseal osteotomy, either using a piezoelectric- (n = 6) or a manual Gigli wire (n = 6) saw. Fractures were stabilized with a Y-shaped mini-locking plate. Within each group, three rats received Alginate directly into the fracture gap. OVX animals gained more weight over 8 weeks compared to CTRL animals. pQCT analysis showed a significant difference in the volumetric cancellous bone mineral density between OVX and CTRL rats. A histological examination of the osteoporotic phenotype was completed. Radiographic evaluation and Masson–Goldner trichrome staining with the piezoelectric saw failed to demonstrate bony bridging or a callus formation. New bone formation and complete healing were seen after 6 weeks in the Gigli group. For the creation of a metaphyseal atrophic non-union in the osteoporotic bone, a piezoelectric saw should be used.

## 1. Introduction

The socio-economic relevance of osteoporosis is steadily increasing, and this metabolic bone disorder is considered the most common, non-communicable bone disease. Diagnosis of osteoporosis relies on the quantitative assessment of bone mineral density, which is a major determinant of bone strength, and the principal clinical manifestation is a fracture [1]. The nonunion of a fracture results in pain, weakness, and reduced mobility especially in geriatric patients [2]. Osteoporotic fractures are particularly challenging to treat due to their decreased bone quality [3], which requires advanced surgical skills to achieve sufficient fracture stabilization. Further, fracture fixation is a limiting factor, as full weight-bearing after reaching surgical reduction for stable fracture retention is often not achievable in geriatric patients [4]. Poor biomechanical properties due to osteoporosis, caused by porous cancellous and thin cortical bone, lead to a higher rate of implant cutout and loosening, as well as peri-implant fractures and the formation of non-unions [5].

To improve the treatment outcome of non-union fractures in addition to its surgical stabilization, preclinical testing of different biomaterials and tissue engineering approaches are on the rise, which are generally evaluated using small animal models [6,7,8].

However, relevant preclinical studies should simulate the clinical setting by introducing a metaphyseal fracture in a relevant osteoporotic animal model. Further, as large animal models are cost-intensive and require specialized housing and surgical facilities, small animal models, e.g., rats, are preferable for proof of concept (PoC) studies. However, the so far published rat models often have several limitations: Drill hole defect models [9,10] are not as clinically relevant as they do not require surgical fixation and underly different healing mechanisms compared to a full fracture. Bilateral osteotomies [11,12] in the tibia or femur have a negative influence on the weight-bearing status and are rarely seen in a clinical setting. Critical size or full triangular defects [13,14] do not resemble fracture patterns frequently observed in geriatric patients and are most suitable to evaluate biomaterials and therapeutic agents for their potential to enhance the repair of large bone defects. Only a few experimental studies on femoral metaphysis with osteotomies in rats have been carried out by Kolios et al. [12], Komrakova et al. [15], Stuermer et al. [16], and Wong et al. [17].

In the literature, there is a lack of clinically relevant osteoporotic models which compare normal femoral bone to osteoporotic bone in the metaphysis of femurs [7,18,19]. Controlled and reproducible fracture-like defects in rat models can either be created with power tools, such as the piezoelectric saw or manual hand tools, i.e., a Gigli saw. As power-tool usage can cause damage to surrounding tissue and structures, such as thermal necrosis [20] and overdrilling/oversawing [21,22], the resulting fracture gap tends to show delayed healing or the formation of an atrophic non-union.

Together, using an in vivo rat femur model with an osteoporotic phenotype as determined using (1) pQCT and (2) histological analysis, we asked: Compared to a manual tool for the creation of an osteotomy, does the use of a piezoelectric saw result in the formation of athropic non-unions categorized by less newly formed bone as determined using (3) radiographic evaluation, and (4) histological evaluation?

Thus, the aim of the current study was to establish a metaphyseal femoral non-union model in osteoporotic rats by comparing a power tool versus a manual tool for osteotomy creation. This model would allow for subsequent testing of new biomaterials to enhance the healing of pseudoarthrosis in osteoporotic bone. Further, this animal model fulfills all criteria to mimic the clinically relevant fracture situation as encountered in geriatric patients.

## 2. Materials and Methods

### 2.1. Experimental Animals

All animal experiments and conducted procedures were in accordance with the Austrian law on animal experimentation and approved by the Austrian regulatory authorities (Permit No. 2020-0.547.757).

A total of eighteen 12-week-old, adult female Sprague Dawley rats (Janvier Labs SAS, Le Genest-Saint-Isle, France) weighing approximately 300–350 g were randomly assigned to each experimental group. In the first experiment, the induction of the osteoporotic phenotype was evaluated over a time course of 8 weeks, comparing ovariectomized rats receiving a special diet (n = 3) to control animals receiving normal rodent chow (n = 3). Subsequently, 12 rats were randomly assigned to the experimental groups outlined under Section 2.2. The animals were kept under standard housing conditions (2–4 rats per cage) with free access to food and water. Post-operatively, the same animals were kept in groups of 2 to 3 rats per cage. Rooms were maintained at 25 ± 2 °C and 12:12 h light/dark cycle, light on at 0700 h.

### 2.2. Animal Study Design

To evaluate the induction of the osteoporotic phenotype, after an acclimatization phase, 6 animals were randomly assigned to be ovariectomized (OVX; n = 3) or to be in the control group (CTRL, n = 3). The osteoporotic phenotype was assessed for 8 weeks using biweekly pQCT measurements, and the animals were then euthanized for histology. Based on the result, 6 weeks post-ovariectomy was chosen for performing the osteotomies in the following experiments, as a robust osteoporotic phenotype was evident with pQCT. In a subsequent experiment, 12 animals underwent OVX surgery followed by femoral surgery 6 weeks post-OVX. The animals were assigned to either the Gigli saw- (n = 6) or piezoelectric saw- (n = 6) induced osteotomy group. Within each group, the osteotomy gap was filled with either alginate (n = 3; PRONOVA SLG-20; NovaMatrix, Sandvika, Norway) or left empty (n = 3) in order to test for the possibility to apply a biomaterial into the gap. All rats underwent radiological and histological evaluation.

### 2.3. Osteoporotic Induction

An osteoporotic phenotype was induced with a ventral ovariectomy in addition to feeding a special diet according to the protocol established by Heiss et al. [23]. Thirty minutes preoperatively, all rats received 0.03 mg/kg buprenorphine as a subcutaneous (s. c.) injection. Anesthesia was induced in an airtight box with 4% (*v*/*v*) isoflurane in oxygen and subsequently was maintained at 2% (*v*/*v*) with a flow rate of 500 mL/min. During the entire procedure, the animals were placed on an electric heating pad to prevent hypothermia (Harvard Apparatus, Holliston, MA, USA). Prior to surgery, each animal received an antibiotic (Clindamycin s.c., 45 mg/kg).

After aseptic preparation, a small transverse peritoneal incision of 0.6–1.0 cm was made on the middle part of the abdomen between the second left and right nipple. After accessing the peritoneal cavity, the adipose tissue was pulled away until the uterine tube and the ovary were identified. The ovary with its associated fat was exteriorized by applying a gentle digital retraction. The same procedure was carried out for the other ovary. A suture (Ethicon non-resorbable suture −4/0, Johnson & Johnson Ltd., New Brunswick, NJ, USA) was set around the area of the distal uterine horn and sectioned thereafter to remove the ovary in toto. The uterine horn was brought back into the peritoneal cavity and the wound was closed in two layers. Post-surgery, all animals received two doses of buprenorphine s.c. (0.03 mg/kg, twice daily, for three days) and tramadol-hydrochloride (20 mg/kg body weight, once daily) in their drinking water for up to 5 days. Wound clips were removed after 7 days. The animals had free access to food and water and were monitored for weight loss or abnormal behavior. CTRL animals did not undergo sham surgery. All animals of the OVX group were fed with a calcium-, phosphorus-, vitamin D3-, soy-, and phytoestrogen-free diet (Altromin-C1034, Altromin Spezialfutter GmbH, Lage, Germany), whereas CTRL animals received a standard diet (ssniff Spezialdiäten GmbH, Soest, Germany).

### 2.4. Quantitative Assessment of the Osteoporotic Bone Phenotype

The development of the osteoporotic status was assessed quantitatively using pQCT analysis (XCT Research SA, Stratec Medizintechnik, Pforzheim, Germany) at biweekly intervals as previously described [24]. The left proximal tibia metaphysis was examined to obtain the volumetric cancellous bone and total bone mineral density of three OVX and three CTRL animals. The observation was carried out for a total of 8 weeks under general anesthesia. At the same time, blood from the vena caudalis mediana was collected from both groups to analyze differences in blood glucose levels with a blood glucose meter (LUNA duo, Wellion-Medtrust GesmbH, Marz, Austria) over time. Glucose parameters have been chosen to exclude excessive weight gain in OVX animals due to metabolic disorders such as diabetes.

### 2.5. Surgical Procedure: Metaphyseal Non-Union Model

**Figure 1:**
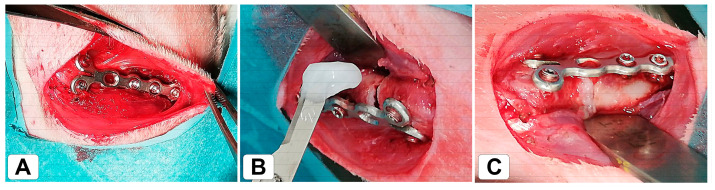
Distal right metaphyseal osteotomy model. (**A**) Macroscopic plate fixation and (**B**,**C**) application of alginate into the fracture gap.

The pre- and post-surgical procedures were the same as mentioned above. Six weeks post-ovariectomy, all OVX rats underwent femur surgery under general anesthesia. After aseptic preparation, a 4 cm skin incision was made on the lateral distal right femur. This was followed by careful deep dissection and coagulation of any veins. Blunt division of the fascia and the vastus lateralis muscle with preliminary preparation directly onto the distal femur. The lateral femur was exposed from the lateral femoral condyle to the lateral midshaft area. The capsule of the knee joint was opened, and the patella was dislocated medially. A plate (Veterinary Orthopedic Implants Inc., 1.5 mm Condylar Angle Stable, DT Locking 2 × 6 hole; Orly, France) was shortened and adjusted with moderate bending. Holes were predrilled with 1.1 mm and the plate was fixed to the lateral femur with two 1.5 mm locking screws with a length of 8.0 mm distally and three 1.5 mm/6.0 mm locking screws proximally (Veterinary Orthopedic Implants Inc.; self-tapping, T4 Star; Orly, France). A distal metaphyseal osteotomy was set with either a piezoelectric oscillating saw (Synthes Piezoelecrtic System, DePuysSynthes; Norderstedt, Germany) with a blade thickness of 0.8 mm or with a 0.66 mm Gigli wire saw (RISystem AG, Lanquart, Switzerland). Subsequently, the site was thoroughly rinsed with a sterile saline solution. According to the experimental treatment groups (see Section 2.2), the osteotomy gap was either filled with an alginate clot (60 µL alginate, 5 µL Ca_2_Cl solution [0.79 g/mL]) or left empty (Figure 1) to test for the possibility of directly applying biomaterials into the gap. Wound closure was performed in layers with the closure of the joint capsule, muscle sutures, and sterile surgical clips (FST, Heidelberg, Germany). Immediately after recovery from anesthesia, the animals were allowed free movement. Immediately post-surgery and at biweekly intervals for 6 weeks, native radiological follow-ups were carried out to investigate the progress of healing.

### 2.6. Histological Examination and Staining

After 6 weeks, the animals were euthanized and the complete right femurs, including muscle tissue, were explanted using exarticulation at the knee and hip joint and fixed in 4% paraformaldehyde (PFA) in PBS. After 48 h at 6 °C, the plates and screws were carefully removed from the samples and afterward decalcified in a 2% PFA/12.5% EDTA solution (pH = 7.5). After a minimum of 7 weeks, the femora were X-rayed again to ensure complete decalcification and processed for paraffin embedding. Then, 6 μm sections were prepared and deparaffinized using Roti^®^-Histol (Carl Roth, Karlsruhe, Germany), rehydrated in a graded alcohol series, and stained with Masson–Goldner trichrome stain [25]. Digital high-resolution images were acquired using a Zeiss Axioplan microscope equipped with an AxioCam MRc5 CCD camera (Carl Zeiss GmbH, Vienna, Austria).

### 2.7. Radiological and Histological Scoring

All radiographs and histological sections were evaluated by two independent observers (AT, AD) who were blinded to the groups. The radiographs were scored for callus formation, quality of union, and bone remodeling as summarized in Table 1 [26,27]. The maximum expected total score is 8 for bone fracture repair. The histological sections were evaluated by the same investigators for fracture healing and scored according to An and Friedman as summarized in Table 2 [28]. The maximum total score for bone fracture healing achievable is 12.

### 2.8. Statistical Analysis

All obtained data samples are reported as means ± standard deviations. Comparison of the gained weight, bone mineral density, trabecular density, and total density between the OVX and CTRL groups were carried out using the Student’s *t*-test. Results of the pQCT were also analyzed using the Student’s *t*-test. Samples were tested for a normal distribution using the Shapiro–Wilk test. Differences in blood glucose levels were evaluated using the Kruskal–Wallis one-way analysis of variance test. Significance was set at *p* = 0.05. All tests were performed using GraphPad Prism v. 9.02 (La Jolla, CA, USA).

## 3. Results

### 3.1. Experimental Results

All OVX animals survived the total observation time of 13 weeks, and no adverse events were recorded. Therefore, all 12 animals were included in this study and evaluated for the metaphyseal non-union osteotomy created using the piezoelectric or Gigli saw. The three CTRL animals were euthanized following the completion of the bone density measurements with pQCT at week 8.

### 3.2. Osteoporotic Induction

**Figure 2:**
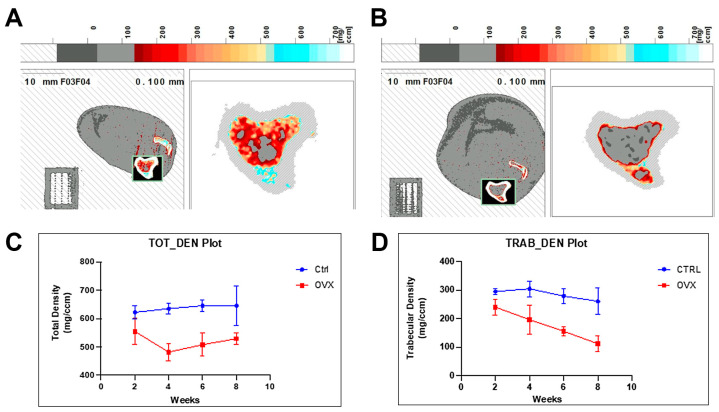
Osteoporotic induction. (**A**) pQCT analysis of the left proximal tibia metaphysis after 8 weeks of non-OVX and (**B**) OVX rats. (**C**) Volumetric cancellous bone mineral density and (**D**) volumetric total bone mineral density in CTRL and OVX rats over 8 weeks (n = 3 per group).

There was a significant difference (*p* = 0.001) in the gained weight between the CTRL animals (141.67 ± 5.84 g) and OVX animals (206.30 ± 23.89 g) over a period of over 8 weeks post-ovariectomy. There was no significant difference in the total baseline metaphyseal bone mineral density between both groups (622.1 ± 24.0 mg/cm^3^ for OVX and 554.6 ± 46.1 mg/cm^3^ for CTRL rats; *p* = 0.0880). pQCT analysis of the proximal tibia metaphysis after 8 weeks showed a significant difference in the volumetric cancellous bone mineral density (Trabecular density/TRAB_DEN) between the OVX and CTRL rats (*p* < 0.0001). TRAB_DEN for the OVX rats revealed a mean of 176.0 ± 57.1 mg/cm^3^ and 284.9 ± 31.5 mg/cm^3^ for the CTRL animals (Figure 2B,D). A significant difference was also observed in the volumetric total bone mineral density (Total density /TOT_DEN) with a mean of 518.2 ± 31.1 mg/cm^3^ for the OVX rats and 637.0 ± 11.1 mg/cm^3^ for the CTRL rats (*p* < 0.0001; Figure 2A,C). Overall, a robust osteoporotic phenotype (Trabecular density with pQCT) was observed after 6 weeks.

**Figure 3:**
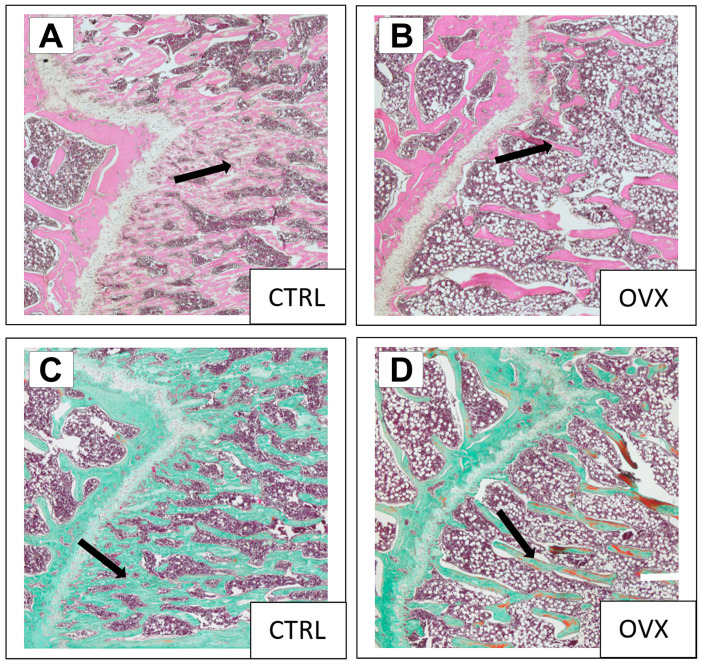
Representative distal metaphyseal femur with H&E staining (top) and Masson–Goldner staining (bottom) confirming the induced osteoporotic bone phenomenon in the OVX group compared to the CTRL group using reduced trabecular density (arrows). CTRL (**A**) and OVX (**B**) animals with H&E staining, compared to CTRL (**C**) and OVX (**D**) rats with Masson-Goldner staining.

Histological examination of the metaphysis in femurs of the CTRL (n = 3) and OVX group (n = 3) 8 weeks post-ovariectomy confirmed the severe osteoporotic phenotype (Figure 3). The monitored blood sugar values at week 0 (129.3 ± 2.5 mg/dL), week 4 (138.7 ± 23.5 mg/dL), and week 8 (129.0 ± 21.1 mg/dL) determined for the OVX animals did not show any significant increase in blood glucose levels over time (*p* = 0.6222; see Table 3).

### 3.3. Radiographic Evaluation

**Figure 4:**
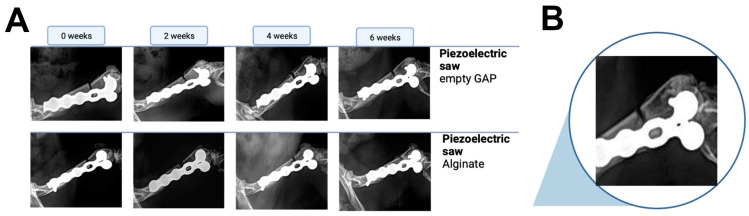
(**A**) Comparison of X-rays left to right from the time of surgery until 6 weeks post-surgery. The top row shows samples without the implantation of an alginate clot, whereas the bottom samples were treated with alginate. (**B**) A close-up of the non-union fracture induced with the piezoelectric saw at 6 weeks post-surgery is shown (representative image of the alginate-treated group).

**Figure 5:**
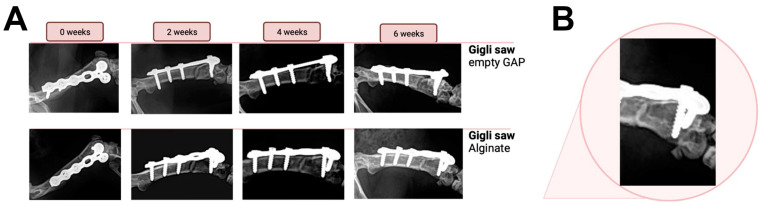
(**A**) Fracture consolidation over a time period of 6 weeks post-surgery after performing a complete osteotomy using a Gigli saw. The top row shows samples without the implantation of an alginate clot, whereas the bottom samples were treated with alginate. (**B**) The fracture gap showed full bridging 6 weeks post-surgery for all animals investigated (representative image of the alginate-treated group).

The obtained X-rays at biweekly intervals were analyzed and documented for comparison. Osteotomies initiated with the piezoelectric saw failed to demonstrate bony bridging or a callus formation in the empty gap or in the defects that had received alginate (Figure 4). A persistent non-union remained in all analyzed animals (n = 3 per group). In comparison, new bone formation and complete healing of the osteotomy were seen after 6 weeks in all animals for which the defect was created with the Gigli wire saw, irrespective of whether they had received alginate or not (Figure 5).

### 3.4. Histology

**Figure 6:**
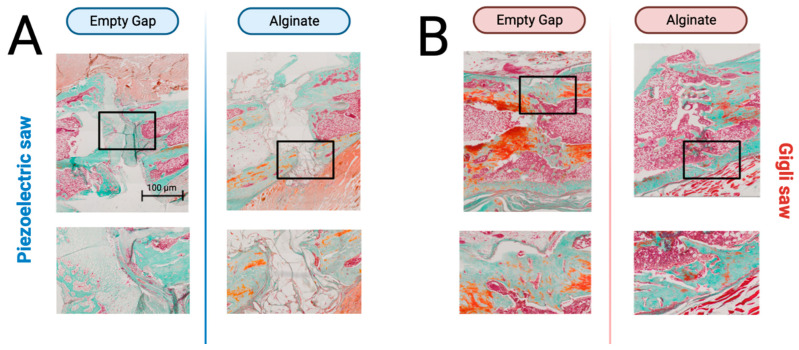
Representative histological sections of the fracture defect area for all treatment groups stained either with Masson–Goldner (bottom) stain. (**A**) Fracture creation with the piezoelectric saw and the (**B**) Gigli saw. The bottom row shows enlarged sections (box) of the fracture defect area.

The histological examination was concurrent with the radiographic findings. A persistent bony non-union zone remained in the gap created with the piezoelectric saw system. There were no signs of callous formation, and overall, the tissue showed all the signs of an atrophic non-union, such as non-mineralized fibrotic tissue, absence of osteotomy bridging, and the rounding of bone ends (Figure 6A). In contrast, osteotomies created using the Gigli saw showed the beginning of bony bridging for all animals investigated during the ongoing bone remodeling. Mineralized bridging zones of the initial osteotomy zone with continuous increasing osseous continuity were observed as well as complete callus formation throughout the process of secondary bone healing (Figure 6B).

### 3.5. Radiological and Histological Scoring

The radiological mean total score was higher in the Gigli group than in the Piezoelectric saw group at the time of termination (6 weeks after femoral surgery). Evaluating all the X-rays, a mean total score of 7 ± 1 in the Gigli group compared to 1 ± 1 in the piezoelectric saw group was determined. Histological scoring yielded comparable results. The mean total histological score was higher in the Gigli group (6 ± 1; n = 6) when compared to the piezoelectric saw group (1 ± 1; n = 6) at the time of termination. It can be summarized that callus formation, cortex remodeling, and bridging at the site of fracture did only occur in the Gigli saw group and could rarely be observed in the piezoelectric saw group. New trabecular bone as well as marrow islands at the periphery of the created fracture were mostly absent in the piezoelectric saw group.

## 4. Discussion

The aim of this present study was to establish a reproducible animal model to evaluate non-unions in osteoporotic bone that is clinically relevant and comparable to encountered pseudoarthrosis patterns frequently observed in the geriatric population.

Non-unions occur in approximately 2% of all encountered fractures [29] but are higher in the diaphyseal area of the femur compared to the metaphysis [30].

This established surgical protocol for metaphyseal femoral non-union allows us to further test novel biomaterials or pro-regenerative factors in a clinically relevant small animal model. The guidelines, as suggested by Alt et al. [14], are fulfilled with this model, with the exception of a critical-size defect: (1) to be carried out in animals with an osteoporotic phenotype and (2) to focus on metaphyseal osteotomy healing. Nevertheless, critical-size defects are rarely encountered in geriatric patients or observed on X-rays after trauma. Therefore, a non-union model seems to be more suitable to allow for further testing of biomaterials in osteoporotic bone. Wong et al. have established a fracture model but do not talk about pseudoarthrosis [17]. In our experimental model, alginate has been chosen because it is an inert material and only for the purpose of demonstrating that the application of a hydrogel to an osteotomy gap is possible, albeit the small dimension of the defect.

As expected, the dietary guidelines established by Heiss et al. [23] promoted the development of a robust osteoporotic phenotype. The pQCT measurements showed significantly lower cancellous and total bone mineral density in the proximal tibia in the OVX group compared to the CTRL group. A sufficient, osteoporotic phenotype along with dietary restrictions has been reached after 6 weeks. Therefore, secondary or follow-up surgeries requiring an osteoporotic bone status can be carried out 6 weeks post-ovariectomy. These results reflect the reproducibility and validity of generating an osteoporotic phenotype.

Bone fractures in geriatric patients mainly occur in the metaphyseal region of long bones [31]. In addition, fractures occur due to bone fragility owing to osteoporosis [32] mainly in the proximal femur and distal radius [33,34,35].

Consequently, it is important to address the metaphyseal and not the diaphyseal region in a preclinical, small animal model to study fracture healing. Several studies have indicated that metaphysis fractures heal via intramembranous ossification in rats [31], but as Inoue et al. demonstrated, ovariectomy itself declines the rate of metaphyseal healing in rats [36] due to delayed inflammation by the late disappearance of neutrophils, decreased formation of medullary callus, and increased endosteal callus formation [12,36]. This emphasizes how vulnerable and concise the process of fracture healing is in rats with a depleted estrogen reservoir. Kolios et al. reported that estrogen improves metaphyseal healing via an increase in the medullary callus formation in OVX rats [11,16,37]. In these experiments, OVX rats had a significant amount of cancellous bone in the metaphysis due to the oral-supplemented estrogen over a 10-week period before the injury.

Osteotomy creation with the Gigli saw showed bony bridging and fracture healing using X-ray analysis after 6 weeks, as well as restoration via secondary bone healing through the formation of a callus and subsequent remodeling. No difference between alginate and the empty gap was observed. In contrast, the X-ray results after osteotomy creation with the piezoelectric saw failed to demonstrate bony bridging or a callus formation in the empty gap or in the alginate group. Although the thickness of the defect created by the two methods varied by approximately 0.1 mm (Piezo vs. Gigli: 0.8 vs. 0.66 mm), it is very unlikely this had a substantial effect on the outcomes as the difference was minimal and both defects cannot be considered a critical-size defect. Nevertheless, we cannot fully exclude that the minor difference in osteotomy thickness also had an impact on the final outcome of this study. 

Piezoelectric osteotomy generates micro-vibrations. Those allow for bone cutting, although the surrounding tissue, including the underlying soft and neurovascular tissue, is not damaged based upon the mechanical effect of ultrasound [37,38,39,40]. Specific to a piezoelectric osteotomy is the cavitation phenomenon. It involves the formation of vapor-filled cavities secondary to the abrupt and rapid changes in pressure in a liquid. The input coming from a high-pressure source causes the collapse of these cavities and generates a shock wave, which propagates in the tissue, ultimately inducing a mechanical cut on the mineralized tissue [41,42,43]. 

We assume that the cavitation effect increased the temperature of the neighboring tissues, although concomitant irrigation should practically lead to the avoidance of thermal injury. This could not be observed in our experiment. We further assume that excessive mechanical vibrations may have altered the bone remodeling cycle, which is already limited in osteoporotic bone. In addition, intra-operative irrigation not only makes the operating site blood-free [44] but also washes out the bone marrow. In comparison, the use of the Gigli saw does not induce any significant increase in temperature and does not require excessive irrigation during the creation of the osteotomy gap. The dispute of overheating and creating a form of pseudoarthrosis, as observed in our model, has been previously described in the literature. Stelzle and Vercellotti reported that an excessive pressure load prevents micro-vibrations in the insert, and the total amount of energy not used for cutting is converted into heat, ultimately leading to damage to the soft tissue and neurovascular structures [45,46,47], hence creating a source of inducing pseudoarthrosis. Indeed, in this study bone formation in the piezo group from the existing cortical bone did not take place as confirmed using the histological evaluation. 

Therefore, the presented metaphyseal non-union model is valuable for testing the application of various biomaterials in pseudarthrosis. This model is a tool to gain insight into the mechanisms favoring a metaphyseal pseudoarthrosis in vivo and replicates a clinical setting more than in previously published critical-size defect models. Consequently, after 6 weeks, the bony fragments in the pseudoarthrotic osteotomy gap should be freshened up and filled with biomaterials or pro-regenerative factors to be tested.

The additional advantage of this metaphyseal non-union model is that the incidence of plate breakage or screw loosening is reduced, as the mechanical forces are most likely lower in comparison to a critical-size defect model, making it more reproducible. 

## 5. Conclusions

The presented model reproducibly induces an osteoporotic phenotype and replicates the clinical situation of pseudoarthrosis frequently seen in the geriatric population. The formation of a non-union in osteoporotic bone with a piezoelectric saw was observed in all treated animals. In contrast, the osteotomy created with a Gigli saw showed complete healing after 6 weeks in X-rays and osseous bridging upon histological evaluation. Therefore, a piezoelectric saw-induced metaphyseal non-union can be considered as a pseudoarthrosis model to evaluate bone healing, e.g., after the application of novel biomaterials or pro-regenerative therapeutic modalities.

## Figures and Tables

**Table 1:** Radiographic scoring system for fracture healing.

Categories	Scores			
	3	2	1	0
Periosteal reaction	Complete across defect	Moderate (>50%)	Mild (<50%)	None
Bone union	Complete bony union	Moderate (>50%)	Mild (<50%)	None
Remodeling		Complete remodeled cortex	Mild (<50%)	None

**Table 2:** Histological scoring system for fracture healing.

Categories	Scores			
	3	2	1	0
Callus formation	Complete across defect	Moderate (>50%)	Mild (<50%)	None
Bone union	Complete bony union	Moderate (>50%)	Mild (<50%)	None
Cortex remodeling	Complete remodeled cortex	Moderate (>50%)	Mild (<50%)	None
Marrow changes	Adult type fatty marrow	Moderate (>50%)	Mild (<50%)	None

**Table 3:** Blood glucose levels over 8 weeks determined for the OVX animals.

Animal	Blood Glucose Levels (mg/dL)		
	Week 0	Week 4	Week 8
OVX1	129	115	131
OVX1	132	162	149
OVX1	127	139	107

## Data Availability

Not applicable.
